# Gas Sensing Properties of Mg-Incorporated Metal–Organic Frameworks

**DOI:** 10.3390/s19153323

**Published:** 2019-07-29

**Authors:** Jae-Hyoung Lee, Thanh-Binh Nguyen, Duy-Khoi Nguyen, Jae-Hun Kim, Jin-Young Kim, Bach Thang Phan, Sang Sub Kim

**Affiliations:** 1 Department of Materials Science and Engineering, Inha University, Incheon 22212, Korea; 2 Faculty of Chemistry, Ho Chi Minh City University of Education, Ho Chi Minh City 721337, Vietnam; 3 Center for Innovative Materials and Architectures (INOMAR), Vietnam National University, Ho Chi Minh City 721337, Vietnam

**Keywords:** Mg-MOF, gas sensor, porosity, toxic gas, sensing mechanism

## Abstract

The gas sensing properties of two novel series of Mg-incorporated metal–organic frameworks (MOFs), termed Mg-MOFs-I and -II, were assessed. The synthesized iso-reticular type Mg-MOFs exhibited good crystallinity, high thermal stability, needle-shape morphology and high surface area (up to 2900 m^2^·g^−1^), which are promising for gas sensing applications. Gas-sensing studies of gas sensors fabricated from Mg-MOFs-II revealed better sensing performance, in terms of the sensor dynamics and sensor response, at an optimal operating temperature of 200 °C. The MOF gas sensor with a larger pore size and volume showed shorter response and recovery times, demonstrating the importance of the pore size and volume on the kinetic properties of MOF-based gas sensors. The gas-sensing results obtained in this study highlight the potential of Mg-MOFs gas sensors for the practical monitoring of toxic gases in a range of environments.

## 1. Introduction

Metal–organic frameworks (MOFs) are a class of hybrid crystalline materials constructed by the appropriate combination of metal clusters and organic linkers that form infinite structures with high porosity, stability, and chemical tenability [1,2,3]. To exploit these advantageous properties, many MOFs have been designed and tailored to a variety of applications, including gas separation and storage [4], conductivity [5], catalysis [6], and drug delivery [7]. For gas sensing applications, MOFs are also a promising material based on their electrical conductivity and adsorption enthalpy for many gases that can be tuned easily [3,4,8].

The high potential of such materials was demonstrated recently for a ZIF-8-coated (ZIF = Zeolite Imidazole Framework) ZnO nanowire (NW) sensor, which exhibited a strong response to H2 in the presence of interfering gases, such as C_6_H_6_ and C_7_H_8_ [9]. In addition, Wu et al. [10] recently reported an excellent selective response of H_2_ in the presence of CO using ZIF-8-coated ZnO nanorod gas sensors. These MOFs act as a type of molecular sieve, which allows only a specific gas molecule to pass through the pores of the MOF used.

On the other hand, specially-designed MOFs can be sensing materials themselves. MOFs with infinite metal chains (-M-O-)_∞_ within the structure, such as the MOF-74 (CPO-27) family of MOFs, are good semiconductors with high charge mobility [11], which makes them a good candidate for gas-sensing applications. Indeed, porous MOFs materials have a high surface area and tunable pore sizes, also making them suitable for gas-sensing applications.

Regardless of the promising potential of such MOFs as gas sensing materials, there are few reports of the sensing capabilities of MOFs, highlighting the need for studies to fill the gap. In this study, two novel MOFs with different pore structures were synthesized: Mg-MOFs-I and -II. Their structures were developed from the MOF-74 form to produce resistance-based gas sensors without any metal oxide carrying agent. The gas sensing applications of the synthesized Mg-incorporated MOFs were investigated systematically. Note that the MOFs without any other metal oxide layer played the role of the active sensing material. The obtained performance of gas sensors fabricated from the Mg-MOFs indicates their potential use in the environmental monitoring of toxic gases.

## 2. Experimental

### 2.1. Chemical

4-aminosalicylic acid (99% purity), terephthaloyl chloride (≥99% purity), magnesium nitrate hexahydrate (99% purity), N,N-dimethylformamide (DMF, ≥99% purity), and N-methyl-2-pyrrolidone (NMP, ≥99.5% purity) were obtained from the Sigma-Aldrich Chemical company (Saint Louis, MO, USA). Oxalyl chloride (98% purity) was acquired from Acros Organic Company. Anhydrous methanol (99.8% extra dry) and ethanol (EMSURE Grade) were purchased from Merck. Finally, diethyl ether (>99.5%, extra dry) was supplied by Fisher Scientific. All other chemicals were purchased from commercial vendors and used without further purification.

### 2.2. Synthesis of Mg-MOFs

H_4_TDA (4,4′-[1,4-phenylenebis-(carbonylimino)]bis(2-hydroxybenzoic acid)]) and H_4_ODA (4,4′-[oxalylbis(imino)]bis(2-hydroxybenzoic acid)]) linkers were synthesized from the N-acylation reactions of 4-aminosalicylic acid with terephthaloyl chloride and oxalyl chloride, respectively. Both Mg-MOFs-I and -II were synthesized through a solvothermal reaction between the H_4_TDA or H_4_ODA linker and the appropriate M^2+^ cation (M^2+^ = Mg), respectively, in a DMF, ethanol, and water (15/1/1, v/v) solvent system according to the process reported elsewhere [12]. Mg-MOFs-I and Mg-MOFs-II synthesized in this study are exactly the same MOFs as Mg-VNU-74-I and Mg-VNU-74-II, respectively, in reference [12]. 

The detail of the synthesis procedure of Mg-MOFs-I is as follows. Mg(NO_3_)_2_·6H_2_O (64.0 mg, 0.249 mmol) and H_4_TDA (37.0 mg, 0.0849 mmol) were dissolved in a 20 mL glass vial containing 7.50 mL of DMF combining with 0.50 mL of ethanol and 0.50 mL of deionized water. The vial was then sealed and sonicated until the solid was completely dissolved. The resulting solution was heated at 120 °C for 48 h in an isothermal oven. Then, colorless, needle-shaped crystals were obtained. The as-synthesized Mg-MOF-I was subsequently washed with DMF (6 × 5 mL) over 2 days before solvent exchanging with dry MeOH (4 × 5 mL, each day for 3 days). The MeOH exchanged samples were activated by the supercritical CO_2_ method using a Tousimis SamdriPVT-3D critical point dryer and heating under reduced pressure (20 mTorr) at 100 °C for 24 h. The detail of the synthesis procedure of Mg-MOFs- II is as follows: Mg(NO_3_)_2_·6H_2_O (87.0 mg, 0.340 mmol) and H_4_ODA (30 mg, 0.083 mmol) were dissolved in a 20 mL glass vial containing 7.50 mL of DMF combining with 0.50 mL of ethanol and 0.50 mL of deionized water, and then heat-treated and activated under the same condition applied to Mg-MOF-I.

### 2.3. Materials Characterization

The morphology of the products was examined by scanning electron microscopy (SEM, Hitachi, S-4800, Tokyo, Japan). The crystallinity of synthesized products was examined by X-ray diffraction (XRD, Bruker D8 Advance, Billerica, MA, USA) using Ni filtered (0.2 mm) CuKα radiation (1.5401 Å). Thermogravimetric analysis (TGA, TA Q500, New Castle, DE, USA) curves were recorded under air flow. The samples were heated to 700 °C at a constant rate of 5 °C/min during all the experiments. Low-pressure N_2_ adsorption experiments were carried out on a Micromeritics 3Flex volumetric gas sorption analyzer. Ultra-pure (99.999%) N_2_ and He gases were used for the adsorption measurements. A liquid N_2_ bath (77 K) was used to take all N_2_ isotherm measurements.

### 2.4. Gas Sensing Tests

**Figure 1:**
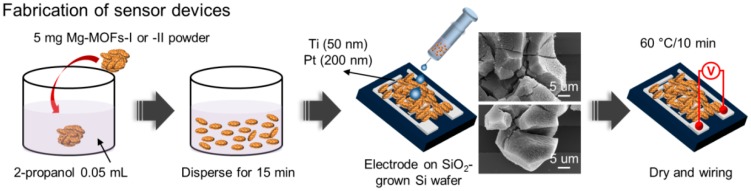
Schematic diagram of the procedure used to fabricate a sensor device with Mg-incorporated metal–organic frameworks (Mg-MOFs).

To fabricate the gas sensors, patterned-interdigital electrodes (PIEs) were prepared on SiO_2_-grown Si (100) substrates using a conventional photolithographic process. Bi-layers of Ti and Pt were deposited sequentially by DC magnetron sputtering. The thicknesses of the Ti and Pt layers were 50 nm and 200 nm, respectively. The Ti layer was used to enhance the adhesion between the SiO_2_ and the Pt layer. The Pt layer served as an electrode. The dimensions of the electrode were 20 µm in width and 1.05 mm in length, the PIE was composed of 20 electrode pads, and the gap between the electrode pads was 10 µm [13,14]. Subsequently, 5 mg of the synthesized Mg-MOFs powders was mixed with 0.05 mL of 2-propanol by ultrasonication for 15 min. A 0.5 μL drop was then taken by micropipette and dropped on the PIEs pattern of the substrate. After drying at 60 °C for 10 min in air, the networked Mg-MOFs were in good contact with the PIEs. Figure 1 shows a schematic diagram of the procedure used to prepare the gas sensor devices with the synthesized Mg-MOFs.

The gas sensing characteristics of the fabricated sensors were examined using a horizontal-quartz heating chamber, where the desired target gas compositions were achieved by mixing an air-balanced target gas and pure dry synthetic air with a total flow of 500 standard cubic centimeter per minute (sccm). Dynamic resistance data were recorded in air (*R_a_*) and in the target gas ambient (*R_g_*), and the response of the gas sensor was defined as *R* = *R_a_*/*R_g_* for NO_2_ gas and *R* = *R_g_*/*R_a_* for the reducing gases. The response time was defined as the time for the sensor to reach 90% of its maximum response after injecting the target gas, and the recovery time was defined as the time taken by the sensor to reach 90% of its initial resistance after the injection of air. Details of the gas sensing tests are reported elsewhere [15,16].

## 3. Results and Discussion

### 3.1. Material Properties of Mg-MOFs

**Figure 2:**
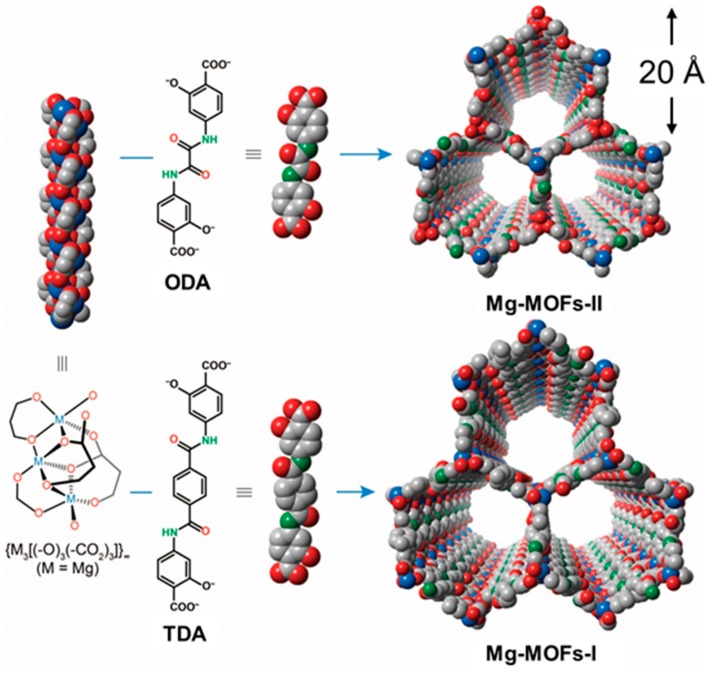
Structures of Mg-MOFs-I and -II. Infinite, rod-shaped metal clusters, {M_3_[(-O)_3_(-CO_2_)_3_]}_∞_ (where M = Mg), are joined with either TDA^4−^ or ODA^4−^ linkers to form Mg-MOFs-I and -II, respectively. C, grey; O, red; N, green, H, pink; Mg atoms, blue. H atoms are omitted for simplicity.

The structures of Mg-MOFs-I and -II contain infinite, rod-shaped secondary building units composed of octahedrally coordinated Mg^2+^ ions and oxygen atoms of the carboxylate and hydroxyl groups of the linkers. They are linked together either by TDA^4−^ or ODA^4−^ linkers (Figure 2). This results in a MOFs structure with a honeycomb shape and an **msg** topology. The pore apertures of one-dimensional, hexagonal channels (i.e., size of the diagonal dimension) of the refined crystal structures of Mg-MOFs-I and -II were calculated to be 26.4 and 22.5 Å, respectively. The amide functional groups from the linkers were aligned along the walls of the channels.

**Figure 3:**
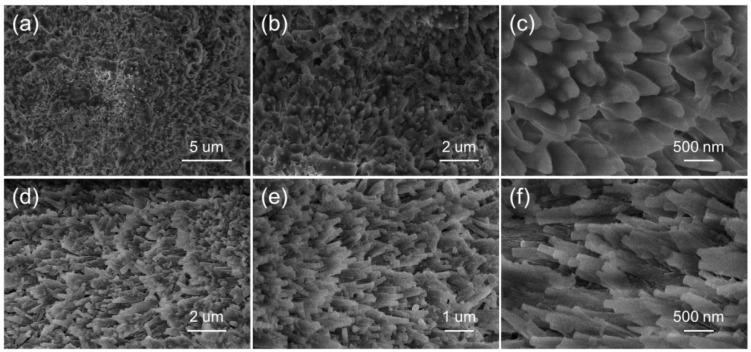
SEM images of (**a**–**c**) Mg-MOFs-I, (**d**–**f**) Mg-MOFs-II.

The morphology of the synthesized Mg-MOFs was examined by SEM, and representative results are shown in Figure 3. Needle-shape particles were obtained in both Mg-MOFs-I and -II samples.

**Figure 4:**
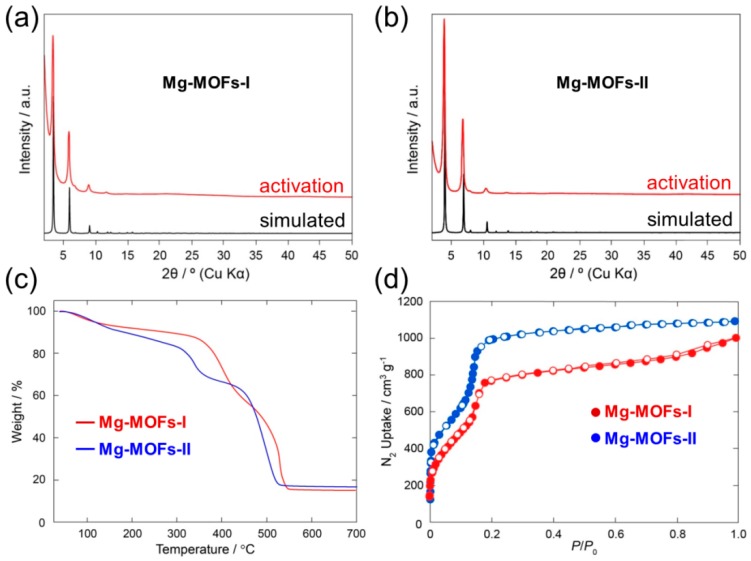
(**a**) XRD patterns of activated (red) Mg-MOFs-I and (**b**) activated Mg-MOFs-II samples (simulated patterns (black) generated from the structural model are provided as a reference). (**c**) TGA curves of (red) activated Mg-MOFs-I (blue) activated Mg-MOFs-II. (**d**) N_2_ isotherms of (red) Mg-MOFs-I and (blue) Mg-MOFs-II at 77 K. The filled and opened symbols represent the adsorption and desorption processes, respectively.

Figure 4a,b show typical XRD patterns taken from Mg-MOFs-I and -II, respectively. Before structural analysis, both were solvent exchanged and then activated to remove the occluded guest species. The experimental patterns were compared with the calculated ones generated from the standard structural models illustrated in Figure 2. Both peaks were well matched with the Mg-MOFs-I and -II structural models, confirming the successful formation of Mg-incorporated MOFs.

TGA (Figure 4c) clearly showed that both MOFs are thermally stable up to ~300 °C. This confirms that the synthesized Mg-incorporated MOFs-I and -II samples were stable at the sensing operation temperatures. To examine the porosity and surface area of the Mg-MOFs-I and -II samples, the N_2_ isotherms at 77 K were obtained, as shown in Figure 4d.

### 3.2. Gas Sensing Properties of Mg-MOFs

**Figure 5:**
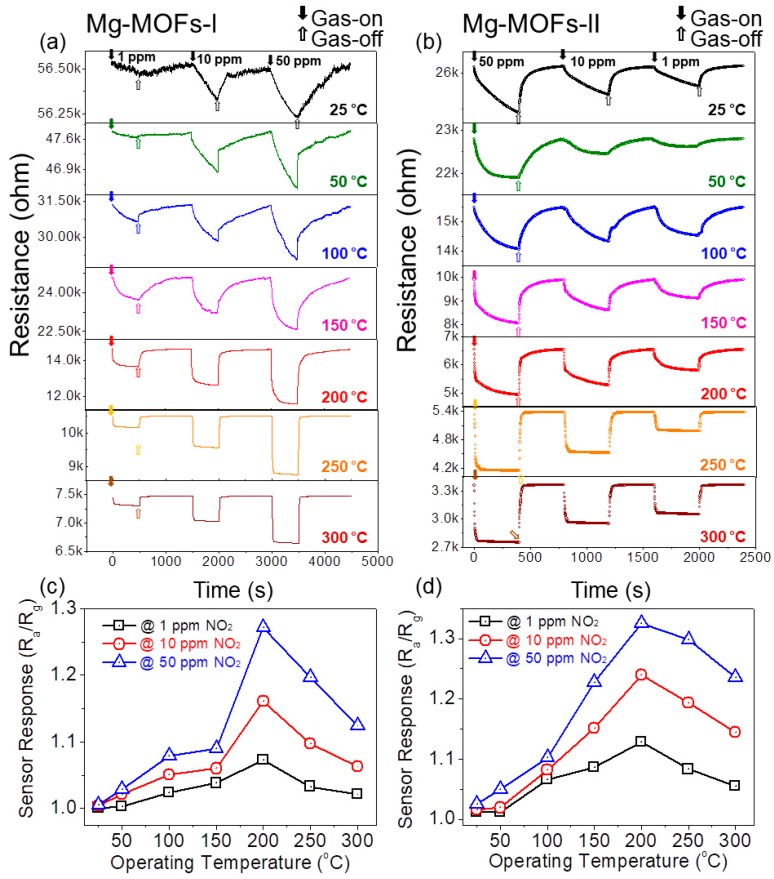
Dynamic resistance curves of (**a**) Mg-MOFs-I and (**b**) Mg-MOFs-II gas sensors towards 1, 10, and 50 ppm NO_2_ gas at different temperatures. Sensor response versus operating temperature at different concentrations for (**c**) Mg-MOFs-I and (**d**) Mg-MOFs-II gas sensors.

The sensing temperature is one of the most important parameters affecting the overall gas sensing behaviors. Therefore, initial sensing tests for NO_2_ gas were performed at different temperatures. Figure 5a,b present dynamic resistance plots of gas sensors fabricated from Mg-MOFs-I and -II, respectively, when exposed to 1, 10, and 50 ppm NO_2_ gas at different temperatures, ranging from 25 to 200 °C. The sensors showed p-type sensing behavior because NO_2_ is an oxidizing gas, and the resistance of the gas sensors decreases upon exposure to the gas. Furthermore, the sensors showed good reversibility because after stopping the NO_2_ gas, the resistance was recovered to their original values. Figure 5c,d show the corresponding response versus sensing temperature curves for Mg-MOFs-I and -II gas sensors, respectively. For both gas sensors, the response increased with increasing NO_2_ concentration because more NO_2_ gas molecules can be adsorbed on the surface of the gas sensors, leading to a stronger response. In addition, the NO_2_ response for both sensors increased with increasing temperature. Indeed, NO_2_ gas molecules require sufficient thermal energy for adsorption, diffusion, and reaction. Hence, higher temperatures provide more energy for the relevant phenomena, leading to a stronger response.

**Figure 6:**
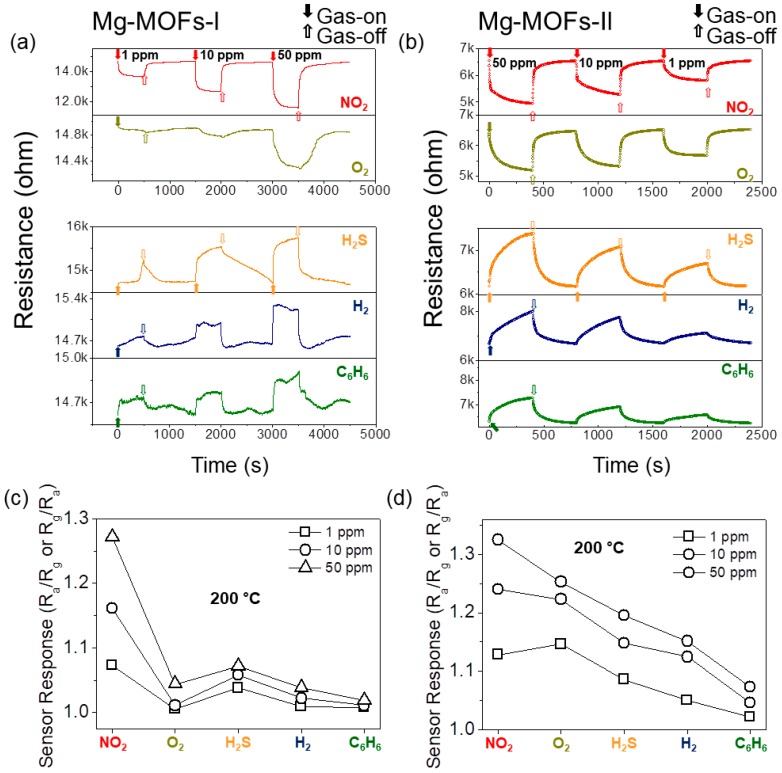
Dynamic resistance curves of (**a**) Mg-MOFs-I and (**b**) Mg-MOFs-II gas sensors towards 1, 10, and 50 ppm NO_2_, O_2_, H_2_S, H_2_, and C_6_H_6_ gases at 200 °C. Sensor response to different gases at 200 °C for (**c**) Mg-MOFs-I, and (**d**) Mg-MOFs-II gas sensors.

Based on the obtained results, the optimal sensing temperature was determined to be 200 °C. In the next step, gas sensors were exposed to different concentrations of various gases, including NO_2_, O_2_, H_2_S, H_2_, and C_6_H_6_. Figure 6a,b show the corresponding dynamic resistance curves for the Mg-MOFs-I and -II gas sensors, respectively. Figure 6c,d present the corresponding response curves at 200 °C for different gases. Both sensors showed a stronger response to NO_2_ than to the other gases.

**Figure 7:**
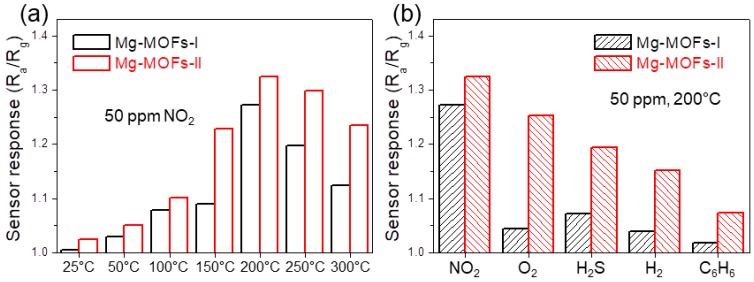
(**a**) Response of Mg-MOFs-I and Mg-MOFs-II gas sensors to 50 ppm NO_2_ gas at various operating temperatures. (**b**) Response of Mg-MOFs-I and Mg-MOFs-II gas sensors to 50 ppm NO_2_, O_2_, H_2_S, H_2_, and C_6_H_6_ gas at 200 °C.

Figure 7a compares the response of Mg-MOFs-I and Mg-MOFs-II gas sensors to 100 ppm NO_2_ gas at various operating temperatures. The NO_2_ response of the sensors improved with increasing sensing temperature. Thermal analysis (Figure 4c) revealed a rapid decrease in weight over 300 °C, suggesting that the synthesized Mg-MOFs decomposed at 300 °C. Accordingly, the maximum sensing temperature was set to 200 °C to maintain the stability of the sensors. Figure 7b compares the response of both gas sensors. The Mg-MOFs-II gas sensor showed a stronger response to all gases than the Mg-MOFs-I gas sensor.

**Figure 8:**
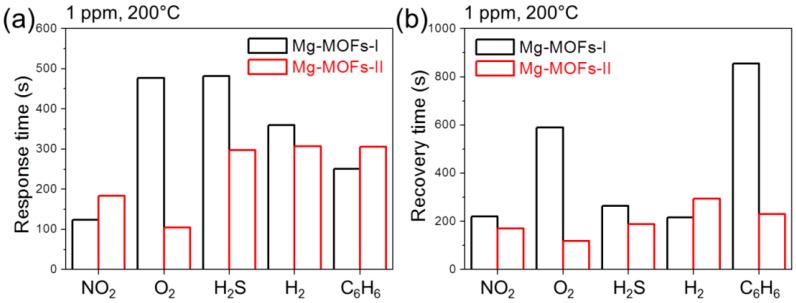
Response (**a**) and recovery (**b**) times of Mg-MOFs-I and Mg-MOFs-II gas sensors for 1 ppm NO_2_, O_2_, H_2_S, H_2_, and C_6_H_6_ gases at 200 °C.

Figure 8a,b show the response time and recovery time of Mg-MOF- I and -II gas sensors to 1 ppm of different gases at 200 °C, respectively. Table 1 lists the response and recovery times of both gas sensors to different concentrations of target gases at 200 °C. In general, the Mg-MOF-II gas sensor showed shorter response and recovery times than the Mg-MOF-I gas sensor. This can be attributed to the larger pore size and volume of the Mg-MOF-II gas sensor.

### 3.3. Gas Sensing Mechanism

**Figure 9:**
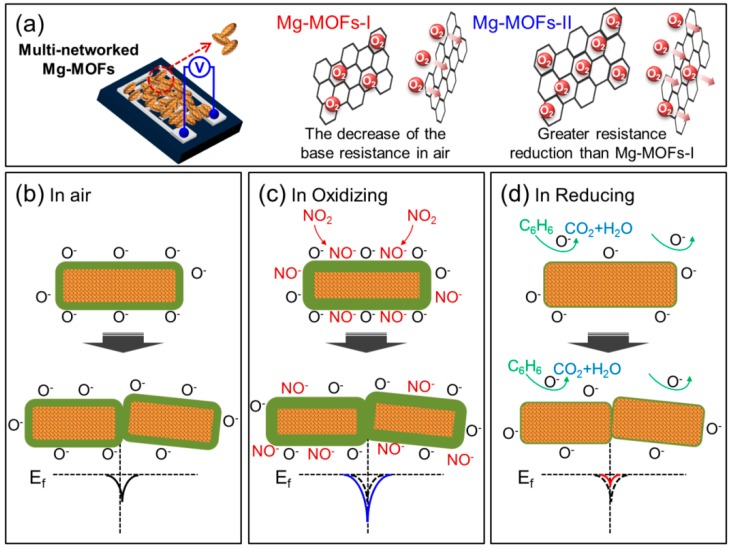
Illustration of the sensing mechanisms operating in Mg-MOFs gas sensors. (**a**) Increase in resistance in the presence of reducing gas (p-type behavior). (**b**) Variation of potential barriers in air, (**c**) oxidizing, and (**d**) reducing gases.

The gas sensors exhibited p-type sensing behavior, where the resistance decreased in the presence of oxidizing gas (NO_2_) and increased in the presence of reducing gases (Figure 9a). In p-type sensing materials, initially in air, caused by the adsorption of oxygen species on the surface of gas sensors, the density of holes increased, and a so-called hole accumulation layer (HAL) was formed on the surface of the gas sensors. In the presence of an oxidizing gas, such as NO_2_, the width of the HAL expands, and the concentration of holes increases relative to electrons by a reaction between NO_2_ and the direct extraction of electrons from the surface of the gas sensors. Accordingly, the resistance of the sensors decreases. For reducing gases, a reverse scenario applies, where the release of electrons to the surface of the gas sensors and the width of the HAL shrinks, consequently the resistance of the sensors increases [17,18,19,20].

Furthermore, the Mg-MOFs-I and -II formed on the electrodes are connected to each other by multiple networking. Accordingly, the variation of potential barriers in different atmospheres should also be considered, as shown in Figure 9b–d. Initially, in air, multiple MOFs are in contact. Potential barriers form among the MOFs, which act as a barrier to charge carriers, due to the adsorption of oxygen species at the contact boundary. When the sensors are in a NO_2_ atmosphere, the height of the potential barriers increases and the variations in the height of the potential barriers modulate the sensor’s resistance due to the abstraction of more electrons from the surface of the gas sensors. This contributes to the response of the gas sensors. On the other hand, when the sensors are in a reducing atmosphere, such as C_6_H_6_, the electrons released return to the surface of the gas sensor and reduce the height of the potential barriers formed among the MOFs, as a result of a reaction between C_6_H_6_ and adsorbed oxygen species, eventually contributing to the sensor’s signal. The selective NO_2_ sensing performance of the MOFs is primarily due to the more reactive nature of NO_2_ gas molecules relative to the other gases tested.

The stronger response of the Mg-MOFs-II gas sensor relative to the Mg-MOFs-I gas sensor can be related mainly to the greater surface area, which is 2900 m^2^·g^−1^ compared to that of Mg-MOFs-I gas sensor (2520 m^2^·g^−1^). A higher surface area leads to more adsorptions sites and consequently, the adsorption of more target gases on the surface of the gas sensors, which results in a stronger change in resistance and corresponding stronger sensor’s response.

Both Mg-MOFs samples have much larger pore sizes (~20 Å) than the kinetic diameters of the gases tested in this study: C_6_H_6_ (5.85 Å), O_2_ (3.46 Å), H_2_ (2.89 Å), H_2_S (3.6 Å), and NO_2_ (3.3 Å) [21,22]. Therefore, the small difference in pore size between the two Mg-MOFs gas sensors is not likely to affect the gas response and selective sensing properties because the flow of gases through the pores in Mg-MOFs is expected to have little effect, and the selectivity of the sensor is thus weak. On the other hand, the response and recovery times appear to be influenced significantly by the pore size and volume in Mg-MOFs. It is reasonable to conclude that the Mg-MOFs-II gas sensor with a larger pore size and volume generally showed shorter response and recovery times. In addition, the base resistance of the Mg-MOFs-II gas sensor was lower due to the greater adsorption of oxygen gas molecules in air. Therefore, it is essential to select a MOF structure with an appropriate pore size and volume when the kinetic properties of gas sensors, such as response and recovery times, need to be considered, particularly when the MOF material is applied directly as a sensing material, not as a sieve. Until now, we do not have enough clear evidences to figure out the role of Mg nodes nor linker structure. Accordingly, further studies to clarify it are necessary in our future works.

## 4. Conclusions

Two novel MOFs, so-called Mg-MOFs-I and -II, were synthesized and used for gas sensing studies. The characterization results demonstrated good crystallinity, good thermal stability, needle-shape morphology, and high porosity of synthesized MOFs. Sensing studies for various gases at 200 °C revealed the Mg-MOFs-II gas sensor to show better performance. In particular, based on the response and recovery times, the size of pores had a stronger impact on the kinetics of the gas sensor than its sieving effect. These results can be used to help design other similar MOF gas sensors with good sensing performance.

## Figures and Tables

**Table 1:** Response and recovery times of Mg-incorporated metal–organic frameworks (Mg-MOFs)-I and -II gas sensors for 1, 10, and 50 ppm NO_2_, O_2_, H_2_S, H_2_, and C_6_H_6_ gases at 200 °C.

**Mg-MOFs-I**										
	**NO_2_**		**O_2_**		**H_2_S**		**H_2_**		**C_6_H_6_**	
	**Res. time (s)**	**Rec. time (s)**	**Res. time (s)**	**Rec. time (s)**	**Res. time (s)**	**Rec. time (s)**	**Res. time (s)**	**Rec. time (s)**	**Res. time (s)**	**Rec. time (s)**
1 ppm	124	221	477	590	482	265	359	216	251	855
5 ppm	80	85	379	461	255	935	36	42	150	123
10 ppm	121	182	233	447	178	303	10	59	460	331
**Mg-MOFs-II**										
	**NO_2_**		**O_2_**		**H_2_S**		**H_2_**		**C_6_H_6_**	
	**Res. time (s)**	**Rec. time (s)**	**Res. time (s)**	**Rec. time (s)**	**Res. time (s)**	**Rec. time (s)**	**Res. time (s)**	**Rec. time (s)**	**Res. time (s)**	**Rec. time (s)**
1 ppm	184	170	105	119	297	189	307	294	306	231
5 ppm	227	129	195	119	278	197	325	227	307	165
10 ppm	167	92	194	94	259	163	321	198	261	175
